# The therapeutic efficacy of denosumab for the loss of bone mineral density in glucocorticoid-induced osteoporosis: a meta-analysis

**DOI:** 10.1093/rap/rkaa008

**Published:** 2020-03-13

**Authors:** Yuta Yamaguchi, Takayoshi Morita, Atsushi Kumanogoh

**Affiliations:** r1 Department of Respiratory Medicine and Clinical Immunology, Osaka University Graduate School of Medicine; r2 Laboratory of Immunopathology, World Premier International Immunology Frontier Research Center; r3 Integrated Frontier Research for Medical Science Division, Institute for Open and Transdisciplinary Research Initiatives, Osaka University, Suita, Osaka, Japan

**Keywords:** denosumab, glucocorticoid-induced osteoporosis, bone mineral density, meta-analysis, anti-RANKL antibody

## Abstract

**Objective:**

Prevention of steroidal osteoporosis is an important issue. There is no clear consensus on the impact of anti-RANKL antibody (denosumab) on BMD in patients with glucocorticoid-induced osteoporosis (GIO). In this study, we aimed to evaluate the impact of denosumab on BMD loss in patients with GIO.

**Methods:**

A comprehensive systematic review and meta-analysis was conducted in accordance with the Preferred Reporting Items for Systematic Reviews and Meta-analyses (PRISMA) guidelines. PubMed, Web of Science and Google Scholar were used to search for original studies reported about BMD in patients with GIO treated with denosumab. In meta-analysis of BMD, the mean difference in the rate of change from baseline and the 95% CI were calculated using the random effects model. The mean differences in patients treated with denosumab were compared with those in patients treated with bisphosphonates.

**Results:**

Out of 713 studies identified, seven studies met the selection criteria for the meta-analysis. At 6 and 12 months of denosumab therapy, increases in BMD were observed in the lumbar spine (2.99% [95% CI 2.71, 3.28] and 4.59% [95% CI 4.17, 5.01]), total hip (1.34% [95% CI 0.64, 2.04] and 2.16% [95% CI 2.05, 2.27]) and femoral neck (0.12% [95% CI −0.38, 0.62] and 1.55% [95% CI 0.45, 2.65]). Additionally, denosumab resulted in significant increases in BMD in the lumbar spine and femoral neck at 12 months compared with bisphosphonate therapy.

**Conclusion:**

Patients with GIO experienced significant increases in BMD in response to treatment with denosumab that were detected in the lumbar spine, total hip and femoral neck at 12 months.

**Key messages**

- Denosumab is an effective treatment for glucocorticoid-induced osteoporosis.
- It may take longer to increase bone mineral density of the femoral neck after denosumab treatment.

## Introduction

Glucocorticoids are important causes of secondary osteoporosis [1]. It is essential to prevent the development of glucocorticoid-induced osteoporosis (GIO) in patients with collagen disease receiving long-term CS treatment. Glucocorticoid-induced loss of BMD is reported to occur 3–6 months after administration of CSs [2, 3]. Therefore, treatment to prevent the reduction of BMD should be considered in the early stage of immunosuppressive treatment.

A bisphosphonate formulation is currently the first choice for prevention and treatment of GIO [4]. Bisphosphonates are easily deposited on bone surfaces and suppress osteolysis by induction of apoptosis and inhibition of enzymes such as farnesyl pyrophosphate synthase (FPPS) in osteoclasts [5]. Although bisphosphonates have been shown to suppress the loss of BMD in patients with GIO [6], some patients are unable to use bisphosphonates because of allergy or side-effects, such as digestive symptoms. Moreover, there is a lack of information on the efficacy and safety of bisphosphonates for long-term usage.

Denosumab is the fully human antireceptor activator of nuclear factor κB ligand (RANKL) antibody; it neutralizes the function of RANKL, which promotes osteoclastogenesis [7]. Denosumab is used to treat postmenopausal osteoporosis and is known to increase BMD in patients with osteoporosis [8]. A recent meta-analysis that included studies of patients with postmenopausal osteoporosis revealed that denosumab promoted more effective increases in BMD than bisphosphonates in the lumbar spine, total hip and femoral neck when evaluated at 12 months after initiation of treatment. Denosumab also decreased the risk of fractures compared with bisphosphonate after 24 months, but this difference was not detected at 12 months [9]. Several studies in patients with GIO have shown that denosumab is also an effective treatment for GIO. Denosumab has been approved for treatment of GIO by the US Food and Drug Administration and the European Medicines Agency since 2018. Yanbeiy & Hansen [10] showed in a meta-analysis that denosumab increased BMD in the lumbar spine and total hip more than bisphosphonates 6 months after treatment initiation in patients with GIO. Although denosumab has been shown to be an effective treatment for patients with GIO, there is little consensus on the amount of increase in BMD from the patient baseline, notably in crucial regions including the lumbar spine, total hip and femoral neck.

To understand the efficacy of denosumab on BMD in patients with GIO in more detail, the present study systematically reviewed original studies of patients with GIO treated with denosumab. Additionally, accumulated evidence on the efficacy of denosumab in patients with GIO was summarized quantitatively by performing a meta-analysis.

## Methods

### Search strategy

Articles documenting the efficacy of denosumab in patients with GIO were examined using three search websites (PubMed, Web of Science and Google Scholar). On PubMed and Web of Science, we performed searching by [(denosumab OR RANKL) AND (glucocorticoid OR steroid OR corticosteroid) AND ‘bone mineral density’]. On Google Scholar, we performed searching by [(steroid-induced osteoporosis) AND (denosumab) AND (bone mineral density OR BMD)]. There were no language restrictions. Data available only in abstracts or unpublished studies were excluded. The searches were performed four times to identify articles published between 1960 and 31 March 2019. Final searches were performed on 2 February 2020. This meta-analysis was performed based on the Preferred Reporting Items for Systematic Reviews and Meta-analyses (PRISMA) statement [11].

### Article selection process

The inclusion criteria were studies of human subjects; original articles (not reviews or case reports); title or abstract including the terms ‘osteoporosis’, ‘steroid’ and ‘denosumab’; available on the Internet; and linkage from the search site to the full text (PDF or website) of the article. Studies that provided no raw data on the mean (s.d.) of the difference in the rate of change of BMD after administration of denosumab were excluded. Redundancies between the PubMed, Web of Science and Google Scholar searches were eliminated (i.e. individual studies were counted only once in this analysis).

### Quality assessment

Two authors (Y.Y. and T.M.) independently checked and selected all references. In the case of inconsistent results, a third person (A.K.) provided an opinion to resolve the issue. The quality of selected studies was assessed according to the Study Quality Assessment Tools (Quality Assessment of Systematic Reviews and Meta-Analyses) from the National Heart, Lung, and Blood Institute (NHLBI) [12]. The evidence level was evaluated based on the Oxford Centre for Evidence-Based Medicine 2011 [13]. Asymmetry of a funnel plot was used to assess publication bias.

### Data extraction

Data were extracted from all studies included in this analysis [author, year of publication, country where the study was conducted, study design (such as cohort or randomized clinical trial), number of patients, age, percentage of females, underlying disease treated with CSs, dosage of CSs, duration of CS usage, BMD, T-score and history of bisphosphonate treatment] and entered into Table 1. To evaluate the effects of denosumab on GIO, the mean (s.d.) of rate of change of BMD at 6 and 12 months after the initiation of treatment with denosumab from baseline (before denosumab treatment) were extracted. When the efficacy of denosumab on BMD among patients with GIO was compared with that of bisphosphonates in the reports included in this analysis, we extracted data on the mean (s.d.) rate of change in BMD from the initiation of treatment with denosumab or bisphosphonates to 12 months. When raw data were unavailable, we calculated values manually using information available in the published graphs and tables.

**Table 1:** Background of patients receiving the treatment of glucocorticoids

Author, year, country	**EL** ^a^ (study design)	Number of patients	Age (years) [mean (s.d.)]	Female (%)	Main conditions of patients	CS dosage (mg/day) [mean (s.d.)]	CS duration [mean (s.d.)]	BMD (g/cm2) Lumbar [mean (s.d.)] Hip [mean (s.d.)] Femoral [mean (s.d.)]	T-score Lumbar [mean (s.d.)] Hip [mean (s.d.)] Femoral [mean (s.d.)]	Treatment history of bisphosphonates (%)
Saag *et al.* [14], 2019, USA	2 (RCT)	253	61.5 (11.6)	73.1	SLE (5.9%) RA (37.9%) PMR (8.3%) Vasculitis (5.9%) COPD (2.8%) Asthma (7.9%) IBD (1.2%)	12.3 (8.09)	0–3 months: 5.1% 3–12 months: 32.0% ≤12 months: 62.5%	ND	−1.92 (1.38) −1.66 (0.96) ND	ND
Iwamoto *et al*. [15], 2018, Japan	3 (cohort)	66	63.4 (12.8)	84.9	SLE (19.7%) RA (37.9%) PMR (14.6%) PM/DM (3.0%) BD (4.6%) Overlap syndrome (12.1%) Others (18.1%)	5.92 (3.79)	11.6 (8.5) years	0.775 (0.195) ND ND	−2.25 (1.64) ND ND	78.8
Iseri *et al.* [16], 2018, Japan	2 (RCT)	14	66.5 (39.0– 75.8)^b^	42.9	SLE (21.4%) AAV (21.4%) MN (14.3%) MCNS (28.6%) FSGS (7.14%) IgA N (7.14%)	5.0 (2.4–8.5)^b^	6.9 (2.2–19.0) yearsb	0.895 (0.787–1.022)^b^ ND 0.672 (0.17)	−1.3 (−2.5–0.3)^b^ ND −1.3 (1.3)	0
Suzuki *et al.* [17], 2017, Japan	3 (cohort)	24	48.4 (1.2)	100	SLE (4.16%) RA (62.5%) PMR (12.5%) PM/DM (0%) MCTD (4.16%) AOSD (4.16%) UC (4.16%) Crohn’s disease (4.16%) After transplantation (4.16%)	5.0 (0.6)	38.1 (5.7) months	0.826 (0.04) 0.549 (0.03) ND	ND (< −3.0 s.d.)* ND	100
Sawamura *et al.* [18], 2017, Japan	3 (cohort)	29	50.4 (15.9)	75.9	SLE (55.2%) RA (20.7%) PM/DM (6.9%) SS (3.4%) BD (3.4%) CKD (10.4%)	7.4 (5.4)	17.4 (9.3), years	ND	ND	62.1
Petranova *et al.* [19], 2014, Bulgaria	3 (cohort)	30	66.7 (7.9)	100	Rheumatic disease (97%)	ND	ND	0.824 (1.16) 0.681 (0.71) ND	−2.95 (0.03) −2.47 (0.23) ND	ND
Mok *et al.* [20], 2014, China	2 (RCT)	21	54.9 (12.8)	100	SLE (81%) RA (19%)	4.6 (2.06)	108.2 (56.0) months	0.833 (0.11) 0.731 (0.09) 0.606 (0.08)	−2.27 (1.02) 1.73 (0.69) −2.19 (0.70)	100

a EL: evidence level was evaluated based on Oxford Centre for Evidence-Based Medicine 2011 [13].

b Median (range from 25th to 75th percentile).

* T-score was described only as <−3.0 (s.d.).

AAV: anti-neutrophil cytoplasmic antibody associated disease; AOSD: adult-onset Still’s disease; BD: Behçet disease; CKD: chronic kidney disease; COPD: chronic obstructive pulmonary disease; FSGS: focal glomerular sclerosis; IgA N: immunoglobulin A nephropathy; MCNS: minimal change nephrotic syndrome; MN: membranous nephropathy; ND: not determined; RCT: randomized clinical trial; UC: ulcerative colitis.

### Data synthesis

A meta-analysis was performed to estimate the efficacy of denosumab in patients with GIO. Clinical data were analysed before and after the initiation of treatment with denosumab or bisphosphonates, and the outcomes were expressed as mean differences and 95% CIs. For all outcomes, the mean differences were calculated using the random effects model (DerSimonian and Laird method) owing to the differences among the studies in the therapeutic protocols and the methods of measuring BMD [21]. Values of *I*^2^ of 25, 50 and 75% were defined as low, moderate and high, respectively [22]. All analyses were conducted using R v.3.5.1 (R project for Statistical Computing) and EZR v.1.29 [23].

## Results

### Study characteristics

**Fig. 1:**
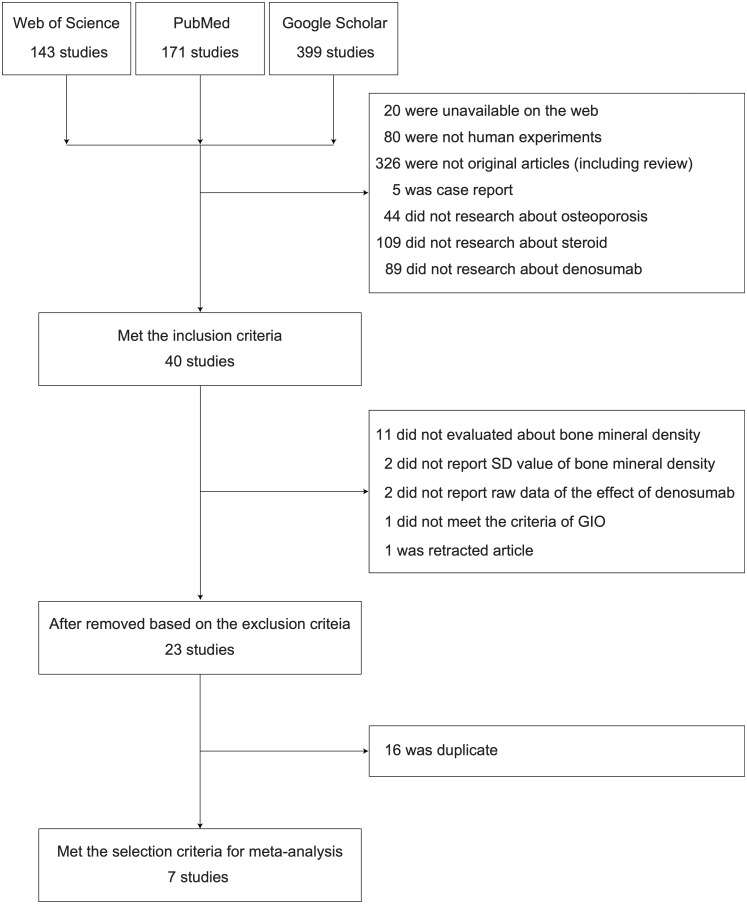
PRISMA flow diagram

We found 171 studies on PubMed, 143 on Web of Science and 399 on Google Scholar. Of these, 673 studies were removed because they did not meet the inclusion criteria based on the title and/or abstract. Subsequently, 17 studies were removed based on the exclusion criteria and 16 because of duplication. Finally, seven studies met the selection criteria for meta-analysis (Fig. 1) [14–20].

The features of the studies included in this meta-analysis are summarized in Table 1. The age of the patients was 48.4–66.7 years, the percentage of females was 42.9–100%, the dosage of CSs was 4.6–12.3 mg/day, and the duration of CS use was 3.17–17.4 years. With regard to the dosage of denosumab, although one study recorded the difference in effect between 60 and 180 mg of denosumab, the other studies administered denosumab at 60 mg. Patient conditions were also described: 19.0–62.5% had RA [14, 15, 17, 18, 20], 4.16–81.0% had SLE [14–18, 20], 8.3–14.6% had PMR [14, 15, 17], 5.9–21.4% had vasculitis [14, 16], 7.14–10.4% had nephritis or chronic kidney disease [16, 18] and 1.2–4.16% had IBD [14, 15, 17].

The BMD before denosumab treatment was 0.775–0.895 g/cm^2^ in the lumbar spine [15–17, 19, 20], 0.549–0.731 g/cm^2^ in the total hip [17, 19, 20] and 0.606–0.672 g/cm^2^ in the femoral neck [16, 20]. Some studies also reported the T-score before treatment; the T-score was −2.95 to −1.3 in the lumbar spine [14–16, 19, 20], −2.47 to 1.73 in the total hip [14, 19, 20] and −2.19 to −1.3 in the femoral neck [16, 20]. The mean (s.d.) of rate of changes in BMD from baseline in the lumbar spine, total hip and femoral neck at 6 and 12 months is summarized in Supplementary Table S1, available at *Rheumatology Advances in Practice* online.

Bisphosphonates were prescribed and evaluated in three studies [14, 16, 20]: risedronate in one study, alendronate in another study and an unidentified bisphosphonate in a third study. The characteristics of patients treated with bisphosphonates in three of the featured studies are shown in Supplementary Table S2, available at *Rheumatology Advances in Practice* online.

The total score of the study quality assessment tools (quality assessment of systematic reviews and meta-analysis) from the NHLBI was 9–11 in each study. As shown in Supplementary Table S3, available at *Rheumatology Advances in Practice* online, points #8 and #12 from the NHLBI study assessment criteria were not met in most of the studies included. Interestingly, much of the data could not be evaluated with funnel plots, which suggests the presence of bias or systemic heterogeneity (Supplementary Fig. S1, available at *Rheumatology Advances in Practice* online).

### Effect of denosumab on BMD of the lumbar spine

**Fig. 2:**
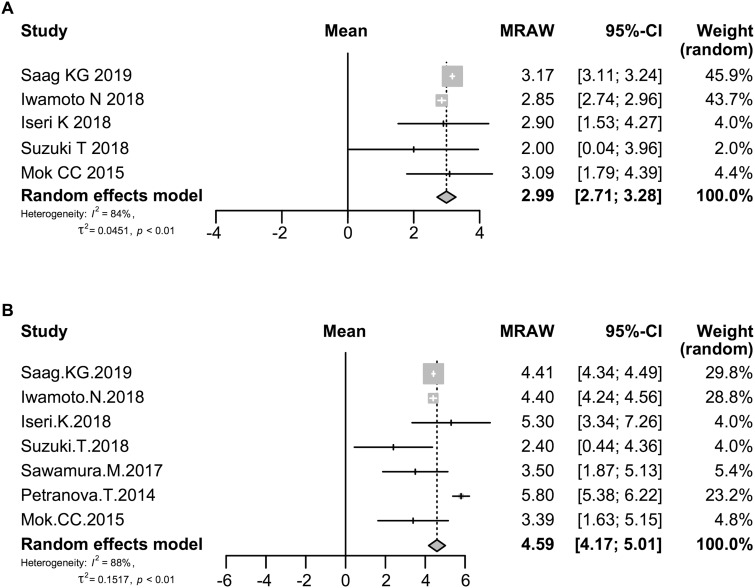
Forrest plot: meta-analysis of BMD in the lumbar spine The rate of change of BMD at 6 (**A**) and 12 months (**B**) after the start of denosumab treatment was calculated using the random effects model. MRAW: raw (untransformed) means.

The effect of denosumab on BMD of the lumbar spine was evaluated in 348 patients at 6 months in five studies [14–17, 20] and in 392 patients at 12 months in seven studies [14–20]. As shown in Fig. 2, the rate of change of BMD of the lumbar spine increased at 6 and 12 months after the start of denosumab treatment by 2.99% (95% CI: 2.71, 3.28; *P *<* *0.0001) and 4.59% (95% CI: 4.17, 5.01; *P *<* *0.0001), respectively. The heterogeneity in BMD of the lumbar spine as assessed by the *I*^2^ statistic was 84% (*P *<* *0.01) and 88% (*P *<* *0.01), respectively.

### Effect of denosumab on BMD of the total hip

**Fig. 3:**
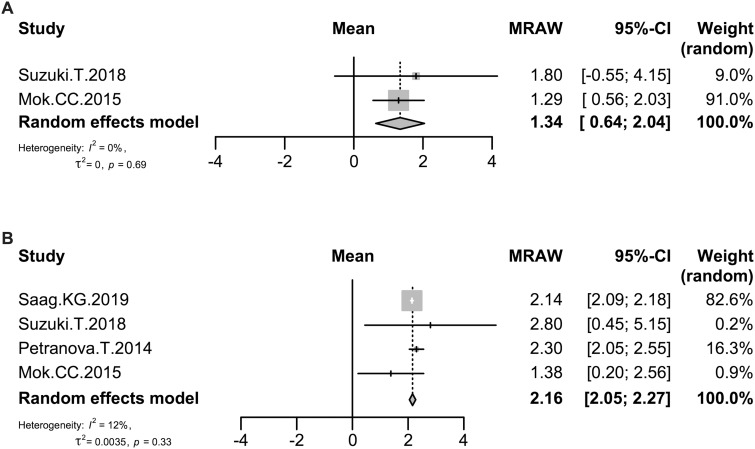
Forrest plot: meta-analysis of BMD in the total hip The rate of change of BMD at 6 (**A**) and 12 months (**B**) after the start of denosumab treatment was calculated using the random effects model. MRAW: raw (untransformed) means.

The effect of denosumab on BMD of the total hip was evaluated in 44 patients at 6 months in two studies [17, 20] and in 291 patients at 12 months in four studies [14, 17, 19, 20]. As shown in Fig. 3, the rate of change of BMD of the total hip increased at 6 and 12 months after the start of denosumab treatment by 1.34% (95% CI: 0.64, 2.04; *P *=* *0.0002) and 2.16% (95% CI: 2.05, 2.27; *P *<* *0.0001), respectively. The heterogeneity in BMD of the total hip as assessed by the *I*^2^ statistic was 0% (*P *=* *0.69) and 12% (*P *=* *0.33), respectively.

### Effect of denosumab on BMD of the femoral neck

**Fig. 4:**
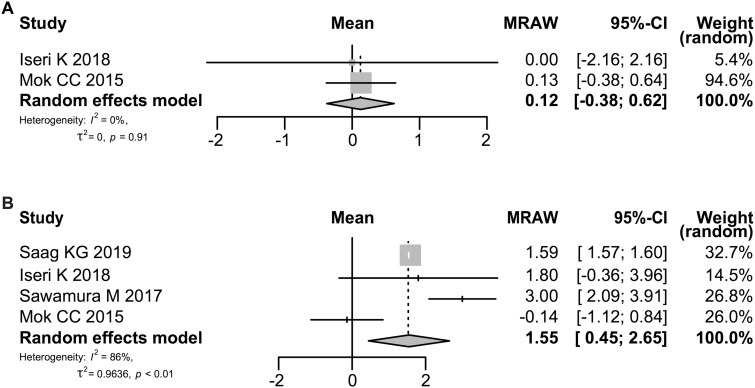
Forrest plot: meta-analysis of BMD in the femoral neck The change rate of BMD at 6 (**A**) and 12 months (**B**) after the start of denosumab treatment was calculated using the random effects model. MRAW: raw (untransformed) means.

The effect of denosumab on BMD of the femoral neck was evaluated in 34 patients at 6 months in two studies [16, 20] and in 280 patients at 12 months in four studies [14, 16, 18, 20]. As shown in Fig. 4, the rate of change of BMD of the femoral neck increased at 12 months after the start of denosumab treatment but not at 6 months; the increase was 0.12% (95% CI: −0.38, 0.62; *P *=* *0.6267) at 6 months and 1.55% (95% CI: 0.45, 2.65; *P *=* *0.0059) at 12 months. The heterogeneity in BMD of the femoral neck as assessed by the *I*^2^ statistic was 0% (*P *=* *0.91) and 86% (*P *<* *0.01), respectively.

### Comparison of the efficacy of denosumab and bisphosphonates on BMD

**Fig. 5:**
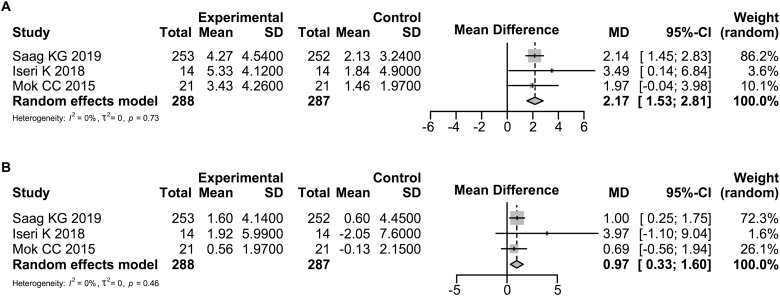
Forrest plot: meta-analysis of BMD between denosumab and bisphosphonates The mean difference in BMD of the lumbar spine (**A**) and the femoral neck (**B**) was calculated using the random effects model. MD: mean difference.

The effect of denosumab and bisphosphonates on BMD of the lumbar spine in 288 denosumab patients and 287 bisphosphonate patients was evaluated at 6 and 12 months in three studies [14, 16, 20]. The characteristics of the patients in these studies are summarized in Supplementary Table S2, available at *Rheumatology Advances in Practice* online. The findings that reveal the rate of change of BMD from baseline in the lumbar spine and femoral neck evaluated at 6 and 12 months after initiating treatment with bisphosphonates are also summarized in this table. The BMD of the lumbar spine in patients treated with denosumab increased at 6 and 12 months compared with that in patients treated with bisphosphonates by 1.43% (95% CI: 0.51, 2.34; *P *=* *0.0023) and 2.17% (95% CI: 1.53, 2.81; *P *<* *0.0001), respectively (Fig. 5A;Supplementary Fig. S2A, available at *Rheumatology Advances in Practice* online). The heterogeneity in BMD of the lumbar spine as assessed by the *I*^2^ statistic was 18% (*P *=* *0.29) and 0% (*P *=* *0.73), respectively.

The effects of denosumab and bisphosphonates on BMD of the femoral neck were evaluated at 6 months in 35 denosumab patients and 35 bisphosphonate patients in two studies [16, 20] and at 12 months in 288 denosumab patients and 287 bisphosphonate patients in three studies [14, 16, 20]. The BMD of the femoral neck in patients treated with denosumab increased at 12 months compared with that in patients treated with bisphosphonates but not at 6 months; the increase was 0.41% (95% CI: −0.65, 1.48; *P *=* *0.4466) at 6 months and 0.97% (95% CI: 0.33, 1.60; *P *=* *0.003) at 12 months (Fig. 5B;Supplementary Fig. 2B, available at *Rheumatology Advances in Practice* online). The heterogeneity in BMD of the lumbar spine and BMD of the femoral neck as assessed by the *I*^2^ statistic was 0% (*P *=* *0.63) and 0% (*P *=* *0.46), respectively.

### Fracture risk

The number of patient fractures observed during the study periods were reported in five studies [15, 17–20]. In two of these studies, no new fractures developed while on denosumab treatment [17, 18]; a third study reported that 3 out of 30 patients developed a new fracture while on this regimen [19]. The risk of fracture in a comparison between denosumab and bisphosphonate therapy was evaluated in three studies [14, 16, 20]. In one study, 36 out of 443 patients sustained fractures among those in the denosumab treatment group as did 36 out of 432 patients receiving bisphosphonate therapy. The relative risk was 0.98 (95% CI: 0.63, 1.52; *P  *=* *0.71). The heterogeneity with respect to the relative risk of fractures as assessed by the *I*^2^ statistic was 0% (*P *=* *0.71) (Supplementary Fig. S3, available at *Rheumatology Advances in Practice* online).

## Discussion

This meta-analysis provides a more definitive insight into increases in BMD from baseline status among GIO patients in response to administration of denosumab. Our findings indicate that denosumab promotes a significant increase in BMD of the lumbar spine, total hip and femoral neck in patients with GIO that can be identified at 12 months after initiation of treatment, although these findings did not include the femoral neck at 6 months of therapy. However, we found that results from denosumab treatment were superior to those from bisphosphonates; denosumab treatment resulted in a significant increase in BMD at the femoral neck that was detected at 12 months. Interestingly, a previous meta-analysis concluded that denosumab had no impact on BMD in the femoral neck at 6 months after treatment compared with results achieved with bisphosphonates [10]. Taken together, these results suggest that improvements in BMD in the femoral neck might require a longer therapeutic course than is required for similar improvements to be detected in the lumbar spine and total hip.

As noted above, the efficacy of denosumab in promoting improvements in baseline BMD differs when comparing results achieved in the lumbar spine, total hip and femoral neck. As such, we propose a hypothesis. First, it is clear that anti-osteoporotic drugs, such as bisphosphonates or denosumab, are effective at promoting increases in BMD in trabecular bone [24]. The proportion of trabecular bone found at target sites is highest in the vertebrae, followed by total hip and femoral neck [25]. Interestingly, the rate of increase in BMD detected in response to denosumab treatment is also highest in the vertebrae, followed by total hip and femoral neck. As such, we hypothesize that differences in trabecular bone mass inherent in each tissue might lead to different rates of response to denosumab.

The femoral neck has a higher percentage of cortical bone than the lumbar spine (75 *vs* 50%) [26]. Cortical bone thickness is reduced in fracture patients compared with non-fracture patients [27]. Therefore, it is important to increase the BMD of cortical bone in order to prevent fractures. In cortical bone, osteoclasts make pores and resorb bone behind the pores. Bisphosphonates tend to be deposited on the bone surface and are less likely to affect osteoclasts behind the pores [28]. Denosumab might suppress the osteolytic function of osteoclasts in cortical bone more than bisphosphonate, because denosumab is an antibody preparation and reaches the back of the pores, where the osteoclasts exist [28].

At this time, little is known about the influence of endogenous CSs on the efficacy of denosumab therapy. Five studies reported that denosumab treatment resulted in increased BMD of the lumbar spine, total hip and femoral neck in patients with postmenopausal osteoporosis at 12 months after the initiation of treatment (by 3.0–5.3, 1.9–3.5 and 1.2–2.4%, respectively) [29]. Although these results cannot be compared directly with our results, the rate of increase in BMD 12 months after initiation of denosumab treatment might be similar to that observed among patients currently on glucocorticoid treatment and those who are not. In patients with GIO, decreased BMD has been observed as early as 6 months after initiation of CS treatment [2]. According to previous reports and consistent with our results, denosumab has more impact on BMD of the lumbar spine and total hip in patients with GIO than bisphosphonates when evaluated at 6 months [10]. As such, introduction of denosumab at an early stage in patients with GIO who have a high risk for fractures might be an important therapeutic option.

It is known that the risk of fracture is higher in patients with reduced BMD than in healthy controls [30]. However, there is no clear consensus on the relationship between the rate of increase in BMD and the rate at which fractures are sustained among patients treated with anti-osteoporotic drugs [31]. Likewise, not only BMD but also bone quality is an important measure of bone strength, although it is difficult clinically to estimate bone quality with current tools available [32]. One study that evaluated the long-term efficacy of denosumab in patients with osteoporosis showed that the rate of increase in BMD was associated with a reduction in risk of fracture in patients with T-scores <−2.0 [33]. As such, it might be clinically meaningful to have a better understanding of the increase in BMD after denosumab treatment in patients with GIO with similar T-scores.

In previous cohort study, we evaluated patients treated with denosumab or alendronate and found no differences in fracture risk over the 3-year observation period [34]. In contrast, denosumab reduced the risk of fracture among patients with postmenopausal osteoporosis compared with what was observed in response to bisphosphonates [9]. The meta-analysis of Yanbeiy & Hansen [10] reported no difference in the risk of fracture among patients with GIO who were treated with bisphosphonate or denosumab; our results are consistent with these findings. As such, we conclude that denosumab might be as effective as bisphosphonates at preventing fracture in patients with GIO. To clarify the efficacy of denosumab for this indication, one would need to examine the impact of this therapy on patients with GIO at high risk of fracture.

Our study has several limitations. First, some of the studies included here did not report sufficient clinical information and provided little information on CS dosages, duration of CS treatment, specific BMD and T-scores for each bone, or any history of treatment with bisphosphonates. In fact, the diseases that triggered the use and dosages of CSs differ between studies. Additionally, it is unknown whether the duration of CS use affects the efficacy of denosumab in patients with GIO. In the future, these problems should be investigated in more detail. Second, it is difficult to deny the influence of the study by Saag *et al.* [14] on the overall result of our meta-analysis despite using the random effect model. Third, our study was based on a population with various backgrounds, such as the underlying diseases, the dosage of CSs or the duration of CS use, resulting in significant heterogeneity. Owing to the limited number of studies and the paucity of clinical data, we were unable to perform a sensitive analysis in detail; thus, the results of the present study should be interpreted with caution. Finally, we have not completely ruled out the possibility of publication bias. Although we created a funnel plot, the results did not generate a symmetrical pattern. The small number of studies and the variation in the number of patients in each study might make it difficult to evaluate the results of a funnel plot.

In conclusion, denosumab therapy resulted in increased BMD in the lumbar spine and total hip in patients with GIO, with positive responses detected at 6 months of treatment; furthermore, increased BMD of the lumbar spine, total hip and femoral neck was evident at 12 months after the initiation of treatment in this patient cohort. Furthermore, denosumab increased BMD of the lumbar spine, total hip and femoral neck more than bisphosphonates 12 months after initiation of treatment in patients with GIO. GIO needs to be prevented completely as an iatrogenic disease. Although there are still problems with denosumab treatment, such as long-term efficacy and complications (risk of atypical fracture and jaw necrosis), we conclude that denosumab is an effective treatment for GIO.

*Funding:* No specific funding was received from any bodies in the public, commercial, or not-for-profit sectors to carry out the work described in this article.

*Disclosure statement:* The authors have declared no conflicts of interest.

## Supplementary data

Supplementary data are available at *Rheumatology Advances in Practice* online.

## Supplementary Material

rkaa008_Supplementary_Data
