# Supplementary material for: The therapeutic efficacy of denosumab for the loss of bone mineral density in glucocorticoid-induced osteoporosis: a meta-analysis
Source: Rheumatol Adv Pract. 2020 Mar 13;4(1):rkaa008. doi: 10.1093/rap/rkaa008 (PMC7197806; doi:10.1093/rap/rkaa008)
Supplement: rkaa008_Supplementary_Data [file rkaa008_supplementary_data.zip › Supplementary Figure Legends.docx]

**Supplementary Figure Legends**

**Supplementary Figure S1. Funnel plot of the effect of denosumab in patients with glucocorticoid-induced osteoporosis.** Each solid circle represents a study. The y-axis represents the standard error that reflects the number of samples, and the x-axis shows the mean rate of change in bone mineral density, which reflects the effect size. Dotted line indicates random effect model estimate and dashed line indicates fixed effect model estimate. The outer dashed lines indicate the triangular region within which 95% of studies are expected to lie in the absence of both biases and heterogeneity.

**Supplementary Figure S2. Forrest plot: meta-analysis of bone mineral density (BMD) at 6 months between denosumab and bisphosphonates.** The mean difference of BMD of the lumbar spine (A) and the femoral neck (B) was calculated using the random effects model. MD: mean difference.

**Supplementary Figure S3. Forrest plot: meta-analysis of relative risk in fracture between denosumab and bisphosphonates.** The relative risk of fracture was calculated using the random effects model. RR: risk ratio.
