# Supplementary material for: The therapeutic efficacy of denosumab for the loss of bone mineral density in glucocorticoid-induced osteoporosis: a meta-analysis
Source: Rheumatol Adv Pract. 2020 Mar 13;4(1):rkaa008. doi: 10.1093/rap/rkaa008 (PMC7197806; doi:10.1093/rap/rkaa008)
Supplement: rkaa008_Supplementary_Data [file rkaa008_supplementary_data.zip › Supplementary Table S1.docx]

|  | Lumbar spine (6 months) | Lumbar spine (12 months) | total Hip (6 months) | total Hip (12 months) | Femoral neck (6 months) | Femoral neck (12 months) |
| --- | --- | --- | --- | --- | --- | --- |
|  | mean (SD) | mean (SD) | mean (SD) | mean (SD) | mean (SD) | mean (SD) |
| Saag KG et al. [17] | 3.17 (0.48), n=224 | 4.41 (0.55), n=209 | ND | 2.14 (0.34), n=217 | ND | 1.59 (0.14), n=217 |
| Iwamoto N et al. [18] | 2.85 (0.47), n=66 | 4.40 (0.67), n=66 | ND | ND | ND | ND |
| Iseri K et al. [19] | 2.90 (2.62), n=14 | 5.30 (3.74), n=14 | ND | ND | 0.00 (4.12), n=14 | 1.80 (4.12), n=14 |
| Suzuki T et al. [20] | 2.00 (4.90), n=24 | 2.40 (4.90), n=24 | 1.80 (5.88), n=24 | 2.80 (5.88), n=24 | ND | ND |
| Sawamura M et al. [21] | ND | 3.50 (4.49), n=29 | ND | ND | ND | 3.00 (2.49), n=29 |
| Petranova T et al. [22] | ND | 5.80 (1.16), n=30 | ND | 2.30 (0.71), n=30 | ND | ND |
| Mok CC et al. [23] | 3.09 (2.96), n=20 | 3.39 (4.02), n=20 | 1.29 (1.68), n=20 | 1.38 (2.68), n=20 | 0.13 (1.17), n=20 | −0.14 (2.24), n=20 |

**Supplementary Table S1. The change ratio from baseline of bone mineral density after administrating Denosumab (%)**

ND, not determined; SD, standard deviation
