# Supplementary material for: The therapeutic efficacy of denosumab for the loss of bone mineral density in glucocorticoid-induced osteoporosis: a meta-analysis
Source: Rheumatol Adv Pract. 2020 Mar 13;4(1):rkaa008. doi: 10.1093/rap/rkaa008 (PMC7197806; doi:10.1093/rap/rkaa008)
Supplement: rkaa008_Supplementary_Data [file rkaa008_supplementary_data.zip › Supplementary Table S2.docx]

**Supplementary Table S2. Patients’ backgrounds and the change ratio of bone mineral density after administrating Denosumab or Bisphosphonates.**

| Author | Treatment | Number of patients | Age (year) [mean (SD)] | Female (%) | Steroid dosage (mg/day)  [mean (SD)] | Steroid duration  [mean (SD)] | BMD (g/cm^2^)/T-score  [mean (SD)] | The change ratio of BMD after treatment (%)  [mean (SD)] | | | |
| --- | --- | --- | --- | --- | --- | --- | --- | --- | --- | --- | --- |
|  |  |  |  |  |  |  | Lumbar spine  Total hip  Femoral neck | Lumbar spine | | Femoral neck | |
|  |  |  |  |  |  |  |  | 6　months | 12 months | 6　months | 12　months |
| Saag KG et al. [17] | Denosumab | 253 | 61.5 (11.6) | 73.1 | 12.3 (8.09) | 0-3 months: 5.1%  3-12 months: 32.0%  12≦ months: 62.5% | ND/−1.92 (1.38)  ND/-1.66 (0.96)  ND/ND | 2.93 (0.53) | 4.27 (0.56) | ND | 1.6 (0.51) |
|  | Resedronate | 252 | 61.3 (11.1) | 73.4 | 11.1 (7.69) | 0-3 months: 3.2%  3-12 months: 29.8%  12≦ months: 66.3% | ND/−1.96 (1.38)  ND/−1.56 (0.96)  ND/ND | 1.87 (0.53) | 2.13 (0.40) | ND | 0.6 (0.55) |
| Iseri K et al. [19] | Denosumab | 14 | 66.5  (39.0-75.8) | 75 | 5.0 (2.4-8.5) | 6.9 (2.2-19.0), years | 0.895 (0.745-1.060)/−1.3 (−2.5-0.3)  ND/ND  0.672 (0.17)/−1.3 (1.3) | 3.20 (0.69) | 5.33 (1.10) | 0.00 (1.01) | 1.92 (1.60) |
|  | Alendronate | 14 | 65.5  (45.0-78.5) | 75 | 5.0 (2.5-9.3) | 9.0 (1.8-19.1), years | 0.875 (0.821-1.045)/−1.2 (−1.9-−0.4)  ND/ND  0.627 (0.11)/−1.7 (0.9) | 1.87 (1.68) | 1.84 (1.31) | −1.33 (1.71) | −2.05 (2.03) |
| Mok CC et al. [23] | Denosumab | 21 | 84.9 (12.8) | 100 | 4.60 (2.06) | 108.2 (56.0), months | 0.830 (0.11)/−2.27 (1.02)  0.731 (0.09)/−1.73 (0.69)  0.606 (0.08)/−2.19 (0.70) | 3.07 (0.64) | 3.43 (0.93) | 0.50 (0.51) | 0.56 (0.43) |
|  | Bisphosphonates  (Unidentified) | 21 | 54.6 (13.4) | 100 | 4.12 (2.14) | 94.1 (75.6), months | 0.810 (0.11)/−2.47 (0.99)  0.748 (0.12)/−1.61 (0.92)  0.625 (0.09)/−2.03 (0.79) | 0.51 (0.60) | 1.46 (0.43) | 0.16 (0.25) | −0.13 (0.47) |

BMD, bone mineral density; ND, not determined; SD, standard deviation
