# Supplementary material for: The therapeutic efficacy of denosumab for the loss of bone mineral density in glucocorticoid-induced osteoporosis: a meta-analysis
Source: Rheumatol Adv Pract. 2020 Mar 13;4(1):rkaa008. doi: 10.1093/rap/rkaa008 (PMC7197806; doi:10.1093/rap/rkaa008)
Supplement: rkaa008_Supplementary_Data [file rkaa008_supplementary_data.zip › Supplementary Table S3.docx]

**Supplementary Table S3. Risk of bias: The Study Quality Assessment from NHLBI**

| Author | Criteria  1^a^ | Criteria  2^b^ | Criteria  3^c^ | Criteria  4^d^ | Criteria  5^e^ | Criteria  6^f^ | Criteria  7^g^ | Criteria  8^h^ | Criteria  9^i^ | Criteria  10^j^ | Criteria  11^k^ | Criteria  12^l^ | Total^*^ |
| --- | --- | --- | --- | --- | --- | --- | --- | --- | --- | --- | --- | --- | --- |
| Saag KG et al. [17] | Yes | Yes | Yes | Yes | Yes | Yes | Yes | Yes | Yes | Yes | Yes | No | 11/12 |
| Iwamoto N et al. [18] | Yes | No | Yes | Yes | Yes | Yes | Yes | No | Yes | Yes | Yes | No | 9/12 |
| Iseri K et al. [19] | Yes | Yes | Yes | Yes | Yes | Yes | Yes | No | Yes | Yes | Yes | NA | 10/12 |
| Suzuki T et al. [20] | Yes | Yes | Yes | Yes | Yes | Yes | Yes | No | Yes | Yes | Yes | No | 10/12 |
| Sawamura M et al. [21] | Yes | Yes | Yes | Yes | Yes | Yes | Yes | No | Yes | Yes | Yes | NA | 10/12 |
| Petranova T et al. [22] | Yes | Yes | Yes | Yes | Yes | Yes | Yes | No | Yes | Yes | Yes | No | 10/12 |
| Mok CC et al. [23] | Yes | Yes | Yes | Yes | Yes | Yes | Yes | Yes | Yes | Yes | Yes | NA | 11/12 |

^*^The total score was calculated based on the study quality assessment tools from the NHLBI [12].

^a^Criteria 1: Was the study question or objective clearly stated?

^b^Criteria 2: Where eligibility/selection criteria for the study population prespecified and clearly described?

^c^Criteria 3: Where the participants in the study representative of those who would be eligible for the test/service/intervention in the general or clinical population of interest?

^d^Criteria 4: Where all eligible participants that met the prespecified entry enrolled?

^e^Criteria 5: Was the sample size sufficiently large to provide confidence in the findings?

^f^Criteria 6: Was the test/service/intervention clearly described and delivered consistently across the study population?

^g^Criteria 7: Where the outcome measures prespecified, clearly defined, valid, reliable, and assessed consistently across all study participants?

^h^Criteria 8: Were the people assessing the outcomes blinded to the participants exposures/interventions?

^i^Criteria 9: Was the loss to follow-up after baseline 20% or less? Were those lost to follow-up accounted for in the analysis?

^j^Criteria 10: Did the statistical methods examine changes in outcome measures from before to after the intervention? Were statistical tests done that provided p values for the pre-to-post changes?

^k^Criteria 11: Were outcome measures of interest taken multiple times before the intervention and multiple times after the intervention (i.e., did they use an interrupted time-series design?)

^l^Criteria 12: If the intervention was conducted at a group level (e.g., a whole hospital, a community, etc.) did the statistical analysis take into account the use of individual-level data to determine effects at the group level?

NHLBI: National Heart, Lung, and Blood Institute
