# Supplementary figures and images for: The therapeutic efficacy of denosumab for the loss of bone mineral density in glucocorticoid-induced osteoporosis: a meta-analysis
Source: Rheumatol Adv Pract. 2020 Mar 13;4(1):rkaa008. doi: 10.1093/rap/rkaa008 (PMC7197806; doi:10.1093/rap/rkaa008)

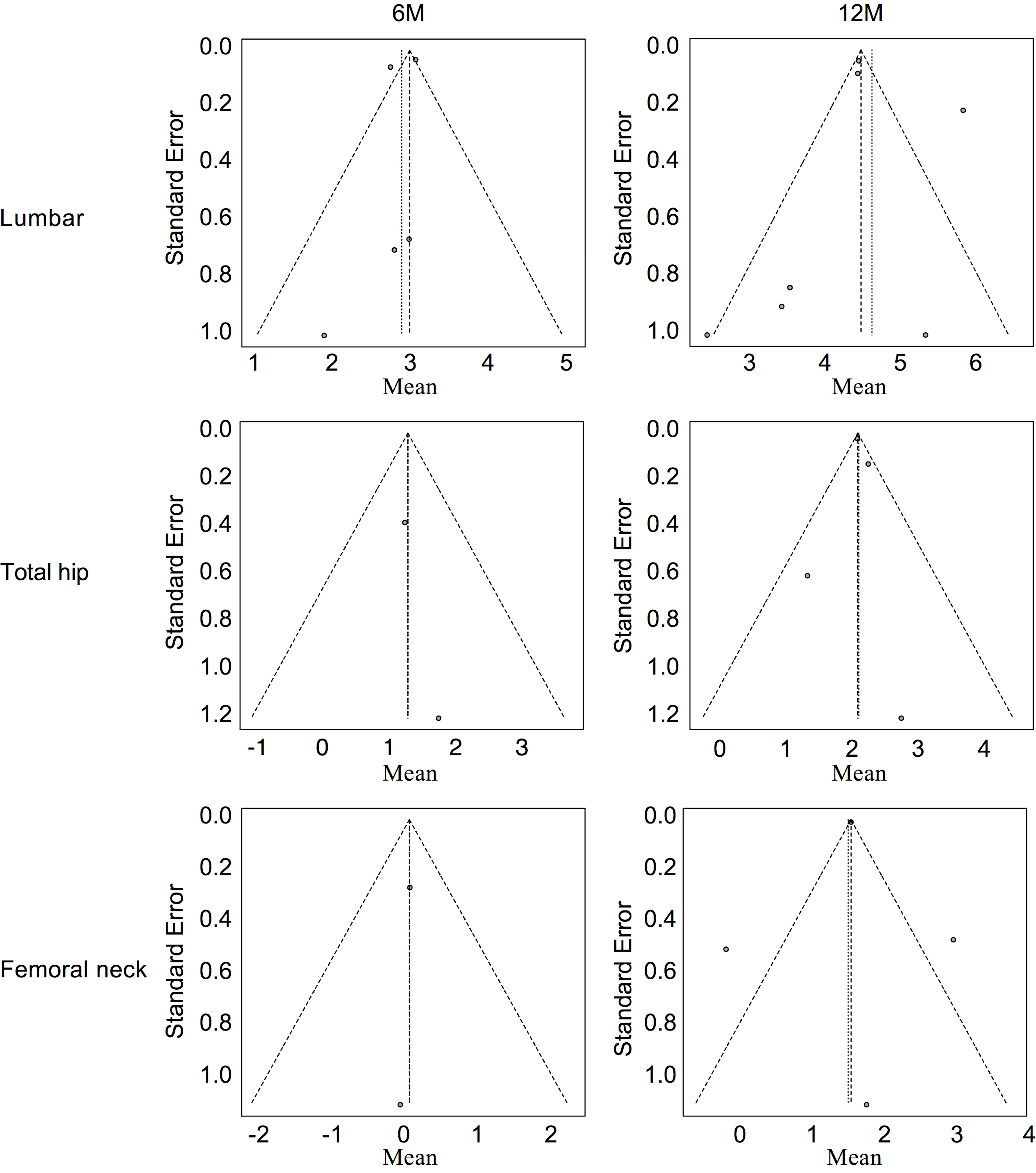

Supplement: rkaa008_Supplementary_Data [file rkaa008_supplementary_data.zip › RAP 20-005.R1 Fig S1.tif]

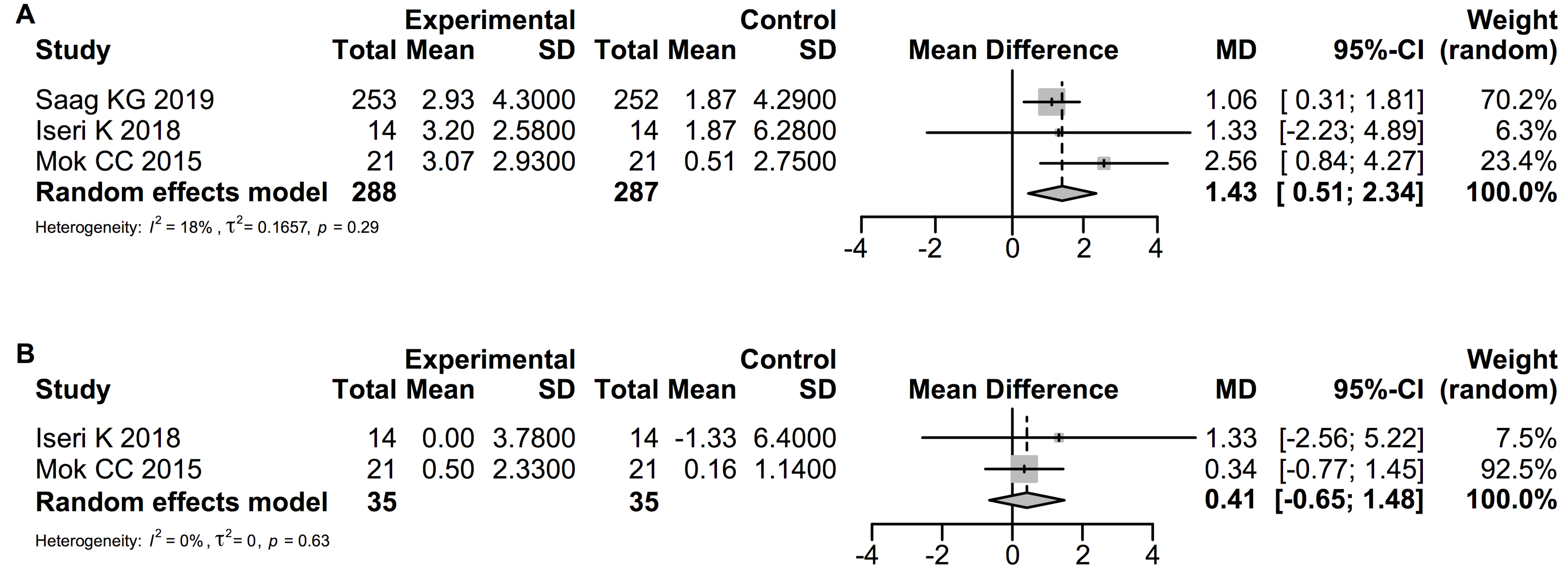

Supplement: rkaa008_Supplementary_Data [file rkaa008_supplementary_data.zip › RAP 20-005.R1 Fig S2.tif]

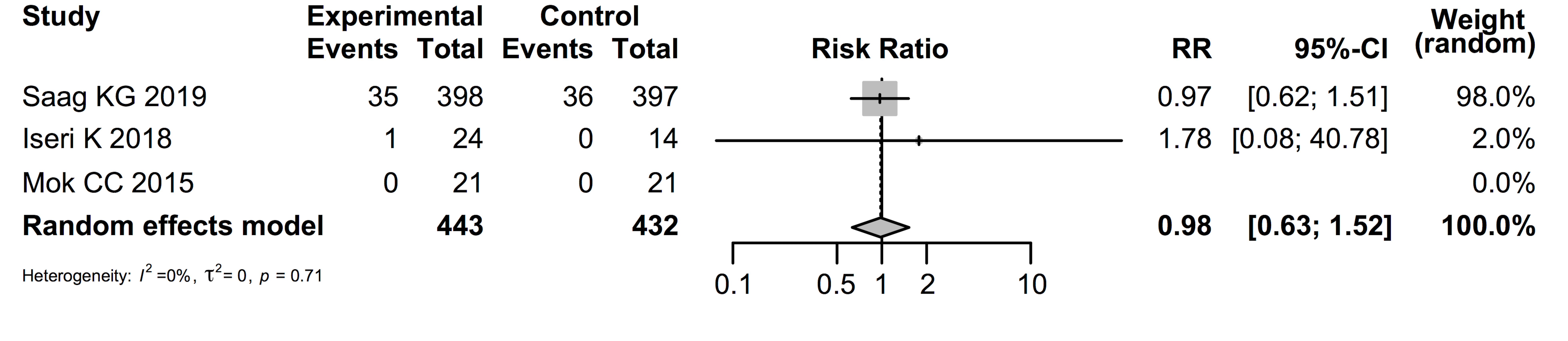

Supplement: rkaa008_Supplementary_Data [file rkaa008_supplementary_data.zip › RAP 20-005.R1 Fig S3.tif]
